# Oncoplastic surgery for the conservative treatment of breast cancer in Perú’s National Cancer Institute

**DOI:** 10.3332/ecancer.2018.815

**Published:** 2018-03-01

**Authors:** Gonzalo Javier Ziegler Rodriguez, Marcelo Diaz Chavez, Gabriela Guadalupe Calderon Valencia, Jose Manuel Cotrina Concha, Milko Raphael Garces Castre, Raul Mantilla

**Affiliations:** 1 Clínica Ricardo Palma, Av Javier Prado 1066, San Isidro 15036, Lima, Peru; 2 Clinica Ziegler, Av Guardia Civil 990, San Isidro 15036, Lima, Peru; 3 Hospital Nivel IV Guillermo Almenara Irigoyen; 4 National Cancer Institute of Perú (INEN); 5 Clínica Montesur; 6 Breast Surgical Oncology Department, National Cancer Institute of Perú (INEN)

**Keywords:** oncoplastic surgery, breast cancer, Latin America

## Abstract

**Background:**

Oncoplastic surgery for breast cancer (OPS) has been a surgical trend for the past 25 years. In 2012, OPS has been introduced as the standard treatment for a selected group of patients at the National Cancer Institute of Peru (INEN). The aim of this study is to describe our findings.

**Methods:**

This is a retrospective and descriptive study that identified demographics, tumour-pathologic features and includes patients solely treated since diagnosis until late follow-up at INEN. These OPS patients were identified from the conservative treatment patients group by review of medical charts and creation of a database for periods December 2005 through December 2015.

**Results:**

A total of 146 patients were ruled in by the inclusion criteria. All patients were Peruvian females, 56.2% being 51 or older. 93.8% had core biopsy diagnosis of breast cancer and 52.1% located at the upper outer quadrant. 79.5% patients had upfront OPS and the round block (43.2%) and reduction/mastopexy (23.3%) were the most used techniques. pT2 was the most frequent size (54.7%). We achieved negative margins in 134 patients (93.2%) in a single procedure. Of 29 patients, who had neoadjuvant treatment, 11 achieved pCR. Only 5.5% had pN2 or higher. 95.2% received complimentary external beam radiotherapy.

**Conclusions:**

OPS has proven to be a reliable surgical option, both for aesthetic and oncologic outcomes. Important points for achieving these results are breast surgeons having properly trained under the OPS philosophy and knowing the patients’ characteristics for correct technique selection.

## Background

Breast surgical oncology has three major precedents in which the European influence has played an important role. In 1878, Halsted travelled to Vienna to learn from the most famous surgeon in Europe, Theodor Billroth [1]. Back in Baltimore, he perfected and popularised ‘the complete method’ obtaining better results than his teacher, reporting 50 breast cancer (BC) surgical cases with only three recurrences, giving way to ‘Halsted’s radical mastectomy’ from 1894 onwards [2].

During the 1970s, Fisher in USA [3] and Veronesi in Italy [4] set the second precedent when exploring other treatment possibilities besides Halsted’s dogma and showed that breast conserving surgery with complimentary whole breast radiation (BCT) had comparable overall survival (OS) and disease-free survival (DFS) versus mastectomy. There was no added benefit in cutting more, so the BCT tendency started [5]. However, BCT still remained as a partial mutilation where asymmetries and deformities were not considered relevant as oncological results were more important than psychological and aesthetic damages. Plastic surgeons developed different ways to handle breast aesthetic problems and soon started using reduction mammoplasty techniques to reshape and reconstruct some BCT cases [6–8] and published different approaches for them [9].

The third event started in 1993 when Galimberti (plastic surgeon) and Veronesi (surgical oncologist) reported a case series in Milan for centrally located tumours where the remodelling of the breast followed the oncologic resection, obtaining acceptable aesthetic results in a single operating day [10]. Audretsch coined the term ‘oncoplastic surgery’ (OPS) for them and it is a surgical philosophy that merges plastic surgery techniques with oncologic clear margins and has gained global acceptance for the satisfaction of patients and their treating surgeons [11]. The French group led by Clough consolidated OPS as a third surgical option for breast surgeons besides BCT or mastectomy. They classified BCT preventable sequelae [12] and developed technical recommendations for OPS by quadrants of the breast. They made it clear that it is a new surgical option for which breast surgeons may receive specialised training [13].

The OPS philosophy was introduced to Latin American countries through specialisation courses led by the Spanish Benigno Acea [14]. Peruvian breast surgeons were no exception. Between 2012 and 2015 breast surgeons including residents and fellows from the National Cancer Institute of Peru (INEN) attended the courses taught by Acea and adopted OPS as a surgical standard for selected patients since 2012. The aim of this study is to analyse a subset of patients treated with OPS in the period 2005–2015 at INEN.

## Methods

From December 2005 until December 2015, more than 1,200 breast conserving surgeries were performed at INEN. We retrospectively reviewed medical charts and built a database for OPS cases. Demographics, tumour characteristics, technical aspects of OPS, axillary surgery, adjuvant therapy, follow-up, and outcomes were collected. We processed information with SPSS v.22 through frequencies, percentages and measures (median, mean and range). Surgeries were carried out exclusively by the breast surgical oncology staff, residents and fellows at INEN properly trained under OPS philosophy. We included patients with confirmed BC diagnosis treated by OPS and received neoadjuvant or adjuvant systemic treatment and excluded patients with bilateral tumours, metastatic disease at diagnosis, patients with inflammatory carcinoma, patients with treatment in a previous institution (surgery, chemotherapy or radiation therapy), patients with incomplete data on medical charts and those lost at sight for more than 6 months in the follow-up appointment. Staging by American Joint Committee on Cancer 7th edition.

## Results

In total, 146 patients met our inclusion criteria. All patients were Peruvian women, where 56.2% of cases were >50 years old. Almost 90% of the cases were diagnosed with biopsy at INEN. About 70% had no co-morbidities. In 52.1% of the cases, the tumour was located at the upper outer quadrant (UOQ), while the least affected was the lower inner quadrant (LIQ). 73.9% of cases had clinically tumours >2 cm (cT2) and 73.6% of cases had clinically negative axilla (cN0). Core biopsy made diagnosis for 93.9% of cases and the rest had fine-needle aspiration (FNA) and cytology, both on an outpatient basis in the clinic. We found 11 *in situ* (10 Ductal carcinoma *in situ* (DCIS), 1 Lobular carcinoma *in situ* (LCIS)) cases in core biopsies but final operative specimen showed nine cases to have infiltrating component. Molecular profiles by immunohistochemistry (IHC) markers were performed in all the core biopsies and in some operative specimens, making a total of 144 analysed infiltrating carcinomas. Luminal A was most frequent (42.2%) and Triple Negative (TN) is less frequent (9%) (Table 1).

**Figure 1.**
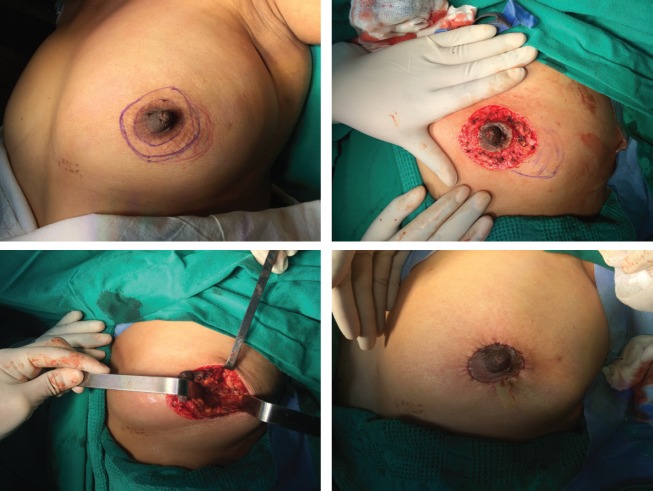
Round block technique. (A) Preop. markings: pattern (blue), tumour (red). (B) De-epithelisation of pattern + tumour marking. (C) Tumour cavity after excision. (D) Final suture + penrose drain (PRD).

**Figure 2.**
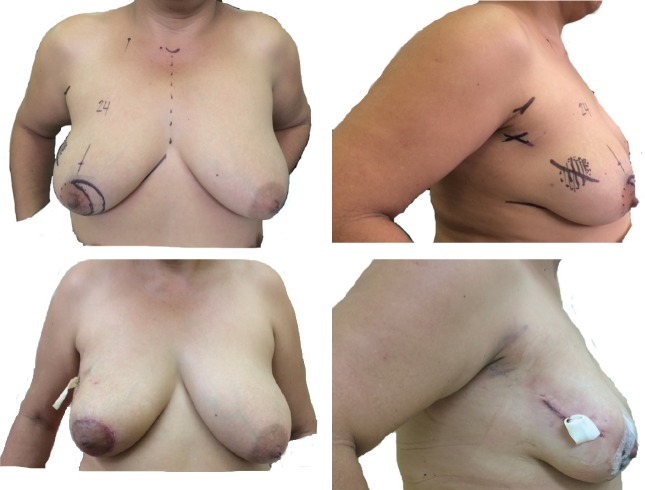
Lateral with re-centralisation of NAC. (A) Preop. Markings of lateral pattern with RNAC (solid line), frontal view. (B) Preop. markings of tumour (dotted line), side view. (c) First day postop, frontal view (with PRD). (D) First day postop, side view (with PRD).

OPS cases were divided into two periods showing only two cases which met our inclusion criteria between 2005 and 2011, whereas the vast majority of OPS was concentrated between 2012 and 2015 with 144 cases. OPS techniques were selected by surgeon discretion depending on the breast size, primary tumour location and level of complexity of the technique (Table 2). Level II techniques were used in all the cases. We identified seven OPS techniques. The most frequent OPS technique was Benelli’s round block (Figure 1) performed in 63 patients (43.2%), followed in frequency by Vertical Mammoplasty (reduction/mastopexy) in 34 cases (23.3%). The Lateral pattern with recentralisation of the nipple–areola complex (RNAC) was an interesting tool for laterally located tumours (Figure 2) and was used in 31 patients (21.2%). Same day symmetrisation of the contralateral side by plastic surgeons was possible in 18 patients (12.3%). Pathology showed that pT2 was the most frequent size (55.4%). 29 cases received neoadjuvant systemic therapy and 11 of them (7.4%) achieved pCR, 5 of which were TN (41.5%), 3 Luminal B (27.3%), 2 Luminal A (18.2%) and 1 case of HER 2 (9.1%).

In two cases (1.4%), the operative specimen showed DCIS in the form of mass >2 cm and other >5 cm. One invasive ductal carcinoma (IDC) case that was diagnosed by incisional biopsy showed no residual disease in the final operative specimen.

We achieved negative margins in 134 patients (93.2%) in a single procedure. Margins could not be defined in 12 cases given that 11 of them had pCR and 1 had incisional biopsy as described. In 124 cases (84.9%), negative margins were obtained after palpable tumour resections. The remaining 10 cases (6.8%) had positive margins, requiring a second surgery with final pathology free of malignancy. No patients had to return to the OR for complications.

Surgical management of the axilla showed 111 sentinel lymph node biopsies (SLNBs) (76%), of which 22 completed axillary lymph node dissection (ALD) (15%). Upfront ALD was done in 35 cases (24%). Final pathology correlated with surgical decision of conserving axillary lymph nodes with 85 cases of pN0 (58.2%) (Table 2).

Over 116 patients underwent upfront OPS (79.5%) and subsequently received the standard adjuvant systemic therapy. Following OPS, 139 patients (95.2%) received complimentary radiotherapy (RT), 112 patients (76.7%) received hormonal therapy and 103 patients (70.6%) received adjuvant chemotherapy (CT) as complementary treatments. Thirty patients (20.5%) had neoadjuvant therapies (Table 3).

In terms of relapses, 141 cases (96.6%) remained disease free while 5 cases showed the following: 1 local recurrence (0.7%), 2 local and distant recurrence (1.4%) and 2 distant recurrences (1.4%). In terms of mortality, 143 cases remained free of malignancy and 3 cases died (2.1%); 2 had received neoadjuvant chemotherapy and then presented with disease progression (one HER 2+ treated without anti-Her2 and one Luminal A case) to bones and lungs, respectively, while the other case died of non-neoplastic causes.

## Discussion

BC incidence is rising globally [15]. Latin American countries like Peru are no exception. The population-based cancer surveillance system of Lima for the period 2004–2005 showed age-adjusted incidence rate (AAIR) of 33.8 cases per 100 thousand women [16], with 3,065 incident cases (only 5.6% of DCIS cases versus USA where DCIS reaches 20–25% [17]). For the period 2010–2012, the registry showed 6,413 incident cases with an AAIR of 40.9 cases per 100 thousand women [18]. Perú´s INEN is located in Lima and records between 2006 and 2015 showed an average of 1,231 new BC cases per year [19].

Since the 1990s [10, 20], the evolution of reconstructive surgical techniques started focusing on BCT deformities [12] and gave way for coupling mammoplasty techniques (during the resective operation) for preventing sequelae using Benelli´s round block [6, 7], vertical patterns like reduction/mastopexy [8], common sense surgical approaches for hidden scars [21] and knowledge about applications and limits of them [14]. All of these contributions have shaped the OPS philosophy to a standarised mapping of the breast by prone-deformity territories and proposing the best OPS technique by tumour/quadrants locations [13, 22, 23]. Even with the well-known utility from neoadjuvant treatment applied to BCT [24, 25], we have avoided resorting to mastectomy and used OPS with presentations like locally advanced disease (taking into account the tumour biology) if a favourable response is achieved and breast conservation can be maximised at its best [26].

Numerous proposals have expanded the OPS range of techniques in the literature [27–39], but it is important to highlight the factors that guide them to success:

- The surgeon must be trained under the OPS theoretical–practical model [40] to understand the importance of breast anatomy and pedicles used by plastic surgery, breast segments proposed by Acea [22] and according to the complexity levels (I–II) proposed by Clough for a proper technique selection that suits the patient and the glandular density of the breast [13, 41, 42].
- The patient must understand the benefits, risks and alternatives of OPS so that they may provide informed consent [43]. This includes an understanding of the co-morbidities and other risk factors like smoking or diabetes [23].

Rietjens *et al* [44] calculated the volume of the operative specimen by multiplying length, width and height. Volume was considered by Garces *et al* [45, 46] and demonstrated that in spite of higher volumetric resections, OPS achieved excellent aesthetics as well as fewer re/excisions for positive margins. Table 4 summarises the versatility of OPS techniques used in European and Latin American publications.

An abstract presented at the annual meeting of the American Society of Breast Surgeons 2017 as a poster by the present article’s authors showed a low number of complications (similar to published literature) for seven OPS techniques performed for both benign and malignant diseases [47]. However in our study, the number of complications was not obtained. We estimate that complications such as seroma, haematoma, dehiscence, and wound infections arose in less than 10% of the cases and were managed on an outpatient basis.

The oncological long-term results of a subgroup of patients in our database are very encouraging with DFS and OS comparable to BCT [48].

## Disadvantages of the study

The 2005–2015 period showed more than 1,206 breast-conserving surgeries in medical charts, of which 806 patients were operated on due to a BC cause. More than 300 cases underwent OPS for different reasons. However, some patients who had received resective surgery previously at another institution with OPS techniques or patients who required complimentary surgery for re-excision or axillary staging were operated on using the OPS approach at INEN, therefore being excluded by our criteria from the study. We also excluded other patients with final pathology reports showing benign tumours (benign Phyllodes, breast lipomas, complex cysts and papillary breast tumours), other malignant non-carcinoma (breast carcinosarcoma) BC, but where the surgical report had missing information about the OPS technique. It is essential to have a standardised perioperative order for the registry of data on clinical charts as well as a surgical report detailing the OPS technique. The technical OPS aspects must be respected to avoid losing valuable information, as well as to report complications in the postoperative follow-up.

## Conclusions

OPS techniques are a new challenge for breast surgeons. Training and proper patient selection are key to maximise aesthetic and oncologic results for the satisfaction of both patients and surgeons.

## Conflicts of interest

None of the authors has any financial interests to disclose.

## Figures and Tables

**Table 1.** Patient demographics, tumour and molecular characteristics.

Characteristic	*N*	%
Gender	F, 146	100
Age		
<35	5	3.4
35–50	59	40.4
51–65	63	43.2
>65	19	13
Previous biopsy		
None	130	89
Incisional, other institution	16	11
Personal history		
None	101	69.2
Comorbidities (high blood pressure, diabetes)	32	21.9
Oncologic		
Breast	4	2.7
Endometrium	3	2.1
Other	6	4.1
Tumour localisation		
UOQ	76	52.1
Upper-inner quadrant	29	19.9
Lower-outer quadrant	20	13.7
Central subareolar	9	6.2
LIQ	6	4.1
≥2 quadrants	6	4.1
Clinical tumour size (cT)		
cT1 (<2 cm)	38	26
cT2 (≥2 cm but <5 cm)	89	60.9
cT3 (≥5 cm)	19	13
Clinical nodal status		
cN0	107	73.3
cN1	32	21.9
cN2a	6	4.1
cN3a	1	0.7
Diagnostic biopsy		
Core	137	93.8
IDC NOS	121	82.9
DCIS	10	6.8
Invasive lobular carcinoma	2	1.4
LCIS	1	0.7
Breast carcinoma, other subtypes	3	2.1
FNA/Cytology: breast carcinoma	9	6.2
IHC molecular profiles for infiltrating tumours*		
Luminal A	61	42.4
Luminal B	42	29.2
Luminal B with HER 2 overexpression	16	11.1
Pure HER 2	12	8.3
Triple negative	13	9

* 2 *in situ* cases were excluded from molecular profiling

**Table 2.** OPS characteristics and axillary management.

Oncoplastic surgery	*N*	%
Cases per period		
2005–2011	2	1.4
2002	35	24
2013	48	32.9
2014	29	19.9
2015	32	21.9
Techniques by frequency		
Round block	63	43.2
Reduction/mastopexy	34	23.3
Lateral with RNAC	31	21.2
Horizontal (Batwing)	6	4.1
Centralectomy	5	3.4
Grisotti technique	3	2.1
Adipofascial rotation flap	3	2.1
Lower-inner quadrant V flap	1	0.7
Symmetrisation	18	12.3
Pathologic tumour size (pT)		
pTis	2	1.4
pT1	51	34.5
pT2	81	54.7
pCR	11	7.4
No residual disease	1	0.7
Margins		
Negative	124	84.9
Not determined	12	8.2
Positive	10	6.8
Axillary management		
SLNB	89	61
SLNB + ALD	22	15
ALD	35	24
Pathologic nodal status		
pN0	85	58.2
pN1 mic	7	4.8
pN1	46	31.5
pN2	7	4.8
pN3	1	0.7

**Table 3.** Oncologic treatment characteristics.

Treatment	*N*	%
Upfront therapy		
OPS	116	79.5
Neoadjuvant CT	28	19.1
Neoadjuvant CT + RT	1	0.7
Neoadjuvant RT	1	0.7
Adjuvant therapy		
CT	103	70.6
Anti-Her2	19	13
Hormonotherapy	112	76.7
RT	139	95.2

**Table 4.** Data comparison from literature between OPS cases reported by different countries.

Author	Year/ country	Period	*N*	OPS	Age	*T*	OPS technique	PO comp	SM
Acea [49]	2005 Spain	2003–2004	160	31%	52.4	T1	46% horizontal	6%	6%
Rietjens [44]	2007 Italy	1994–1999	148	100%	50	T1	Vertical, round block	11%	0.7%
Garces [45]	2013 Peru	2012	78	100%	58	T2	40% vertical	NR	0.4%
De Lorenzi [50]	2015 Italy	2000–2008	454	33.3%	<50	T1	30% vertical	10.3%	15.4%
Clough [26]	2017 France	2004–2016	350	100%	57	T2	41% lateral	8.9%	8%
Ziegler	2017 Peru	2005–2015	146	100%	51	T2	43.2% round block	NR	2.7%

OPS: % operated by OPS from the population; Age: mean age at surgery; T: Tumour size most frequently found; OPS technique: most frequently resorted OPS; PO comp: postop complications, NR: not reported; SM: salvage mastectomy.
